# Entropy-driven formation of chiral nematic phases by computer simulations

**DOI:** 10.1038/ncomms11175

**Published:** 2016-04-12

**Authors:** Simone Dussi, Marjolein Dijkstra

**Affiliations:** 1 Soft Condensed Matter, Debye Institute for Nanomaterials Science, Utrecht University, Princetonplein 5, 3584 CC Utrecht, The Netherlands

## Abstract

Predicting the macroscopic chiral behaviour of liquid crystals from the microscopic chirality of the particles is highly non-trivial, even when the chiral interactions are purely entropic in nature. Here we introduce a novel chiral hard-particle model, namely particles with a twisted polyhedral shape and obtain a stable fully entropy-driven cholesteric phase by computer simulations. By slightly modifying the triangular base of the particle, we are able to switch from a left-handed prolate (calamitic) to a right-handed oblate (discotic) cholesteric phase using the same right-handed twisted particle model. Furthermore, we show that not only prolate and oblate chiral nematic phases, but also other novel entropy-driven phases, namely chiral blue phases, chiral nematic phases featuring both twist and splay deformations, chiral biaxial nematic phases with one of the axes twisted, can be obtained by varying particle biaxiality and chirality. Our results allow to identify general guidelines for the stabilization of these phases.

## Abstract (web summary)

The entropy-driven formation of cholesteric liquid crystal, one of the widely used liquid crystal phase, has not yet been addressed in simulations due to large unit cells. Here, Dussi and Dijkstra overcome this problem by introducing a hard-particle model and show that shape alone can stabilize a chiral nematic phase.

## Graphical abstract

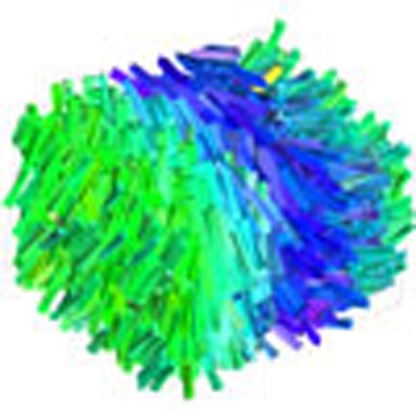

Since Onsager's prediction of a purely entropy-driven phase transition from an isotropic fluid of infinitely long hard Brownian rods to an orientationally ordered nematic phase^1^, hard particles have served as paramount models in condensed matter studies. The seminal work on crystallization of hard spheres revealed the crucial role of computer simulations in proving that order can be induced by entropy alone^2^,^3^. The macroscopic structure obtained by self-assembly of colloidal particles is often directly linked to the shape of the constituent building blocks^4^,^5^. As soon as we move away from spherical particles a bewildering variety of thermodynamically stable structures with increasing complexity arises^4^,^6^. As a result, a concurrent increase of simulation studies on hard particles show that entropy can be the sole driving force in the formation of crystals featuring different symmetries, plastic crystals, liquid crystals and even quasi-crystals^4^,^6^,^7^,^8^,^9^,^10^,^11^,^12^,^13^,^14^.

Shape anisotropy is the essential ingredient to form liquid crystals (LC), phases featuring long-range orientational order but no or only partial positional order^15^. Hard bodies have been extensively employed also in the field of liquid crystals^16^. Thirty-five years after Onsager's prediction, the first entropy-stabilized nematic phase was observed in computer simulations of hard ellipsoids^7^. In the nematic liquid-crystalline phase, the particles are on average aligned along a preferred direction, identified by the nematic director 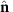, but the positions are homogeneously distributed in the system. Additionally, hard spherocylinders were employed in simulations to demonstrate the thermodynamic stability of an entropy-driven smectic phase^8^, in which the particles are orientationally ordered and arranged in smectic layers. This system has become a popular hard-particle model system to study liquid crystal phase behaviour^17^,^18^. By introducing biaxiality in the hard-particle shape, the long-searched biaxial nematic phase has also been simulated^9^,^19^. Furthermore, many other liquid crystal phases have been observed in simulations, which are entropy driven, including a cubic gyroid phase^20^ and a twist-bend nematic phase^21^.

Surprisingly, from this long list of entropic liquid crystal and non-liquid crystal phases, a simulation evidence of a cholesteric phase made of hard particles is still missing, despite the facts that it was the first liquid crystal phase experimentally discovered^22^ and that an entropic cholesteric phase was already theoretically predicted 40 years ago^23^. A cholesteric phase displays an helical chiral arrangement of the director field, 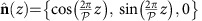, with *z* the axis of the macroscopic twist (chiral director) and 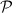 the cholesteric pitch that determines the typical length scale associated to the helical periodicity. Several theoretical studies have been dedicated to better understand the link between microscopic and macroscopic chirality. A unified picture has still yet to be achieved since it is clear that the cholesteric pitch 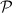 depends in a non-trivial way on both the single-particle properties and the thermodynamic state of the system (for example see refs 23, 24, 25, 26, 27, 28). The microscopic origin of chirality has also been the focus of experimental studies on colloidal systems^26^,^29^,^30^, and of computer simulation studies based on strongly chiral attractive interactions^31^,^32^,^33^.

The main reason that a cholesteric phase of hard bodies has never been observed in simulations is due to the fact that the cholesteric pitch length is on the order of hundreds or thousands times the particle length, and that huge system sizes, beyond our computational limits, are needed to accommodate the cholesteric pitch. Hence it is still unsettled if and how a twist in the particle shape gives rise to the cholesteric order and several questions, that have been addressed by computer simulations for the achiral nematic phase, like nucleation, interfacial and wetting behaviour^18^,^34^, remained so far unexplored for the cholesteric phase. Here, we show the first fully entropy-driven cholesteric phase obtained by computer simulations of hard twisted polyhedral shaped particles.

## Results

### A novel chiral hard-particle model

Recently, hard helices have been introduced as a simple particle model, but the formation of a cholesteric phase has never been addressed in simulations^12^,^35^. Moreover, these simulations showed that systems of hard helices are largely affected by slow equilibration due to an intricate coupling between translational and rotational degrees of freedom, and reveal a novel screw-like nematic phase, in which the helices are interlocked. In addition, theoretical predictions showed that only helices in a very narrow shape parameter range exhibit a cholesteric pitch that becomes accessible in simulations^28^,^36^,^37^. To support these theoretical predictions, we demonstrate the formation of a cholesteric phase of hard helices for which the cholesteric pitch is sufficiently small in Supplementary Fig. 1. However, for these helices the stability density regime of the cholesteric phase is also very narrow^12^,^35^, which makes it hard to perform a thorough investigation on the density dependence of the cholesteric pitch and the effect of particle chirality and particle biaxiality on the chiral nematic phase. To alleviate problems of slow equilibration due to particle entanglement in the case of helical or grooved particles, and cholesteric pitch lengths that exceed our computational limits, we resort to a novel type of particle shape, namely hard twisted polyhedra. The advantage of polyhedral shaped particles is that these particles tend to align their flat faces, thereby speeding up the equilibration, whereas hard helices get easily entangled.

**Figure 1:**
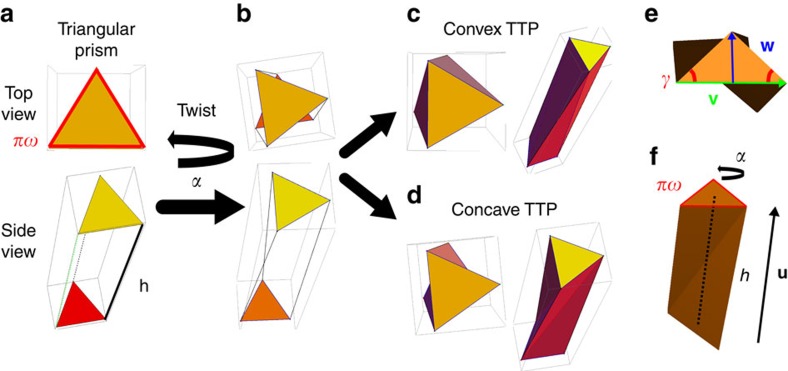
Twisted triangular prism model. (**a**) A twisted triangular prism (TTP) is constructed from an elongated prism of height *h* with (isosceles) triangular bases, determined by the base angle *γ*, and perimeter *πω*. The width *ω* is used as the unit of length. (**b**) To introduce chirality, one triangular base is twisted by a twist angle *α* relative to the other one and additional edges are constructed to obtain flat faces. Depending on the choice of these additional edges, it is possible to build both (**c**) convex and (**d**) concave chiral particles. (**e**,**f**) The TTP is described by the vectors **u**, **v** and **w**, and the orientations of the particle axes are described by the unit vectors 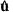 (long), 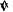 (medium) and 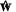 (short).

As we explain below, this particle model presents several shape features that can be easily tuned, e.g., aspect ratio, convexity, biaxiality, handedness, degree of twist (or molecular pitch) and number of polyhedral faces. A systematic study of how these properties, some of which are intuitively associated to microscopic chirality and liquid-crystalline behaviour, affect the self-assembly of many of such particles, can be efficiently carried out by performing computer simulations. In particular, here we study the nematic phase behaviour of the simplest shape of this class, i.e., twisted triangular prisms (TTP). Our particle is obtained by twisting one base of an elongated triangular prism of height *h* by an angle *α* relative to the other base and by adding additional edges to ensure flat faces (Fig. 1). Remarkably, depending on the choice of which vertices are connected by these additional edges, it is possible to build both concave and convex chiral particles. The triangular base has fixed perimeter *πω*, such that in the limit of infinite number of sides (circle) the width *ω* coincides with particle diameter. In this study we consider concave TTPs with either equilateral or isosceles triangular bases defined by the base angle *γ*. When the top triangular base is rotated clockwise the twist angle *α* is positive and it is tempting to call the TTP right handed. We return to this definition of particle handedness when we discuss our results for the oblate (discotic) cholesteric phases. We note that *α* should be less than or equal to the smallest angle of the base to avoid self-intersection of the particle shape. For convenience, we also introduce the vectors **u**, **v** and **w** describing the TTP as shown in Fig. 1, which allow us to define the long, medium and short dimensions of the TTP by their length |**u**|, |**v**| and |**w**|, whereas the particle frame is described by the long, medium and short particle axis denoted by the unit vectors 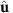, 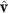 and 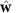. Note that the height *h* coincides with |**u**|. To detect overlaps between particles, i.e., the key ingredient in Monte Carlo (MC) simulations aimed to study the self-assembly of hard particles, we implement an algorithm based on triangle–triangle intersection detection using the RAPID library (http://gamma.cs.unc.edu/OBB/), which is also suitable for concave shapes. Analogously to spherocylinders^17^ (and other hard-rod models), the nematic phase can be stabilized at sufficiently high aspect ratio (*h*/*ω*), whereas the particle chirality can be tuned by changing the twist angle *α* that also changes the molecular pitch 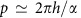. Additionally, by further modifying the particle shape (changing the base) we study how the competition between biaxiality and chirality propagates from microscopic (single particle) to macroscopic (self-assembled structure) level.

Nematic phases formed by biaxial particles can be distinguished in prolate (calamitic) _+_, oblate (discotic) _−_ and biaxial _*b*_ phases, depending on which particle axes feature long-range orientational order. By introducing a shape parameter based on the dimensions of the particle *ν*=|**u**|/|**v**|−|**v**|/|**w**|, the type of nematic phase can roughly be predicted^9^,^38^: For *ν*>0, a prolate _+_ phase is expected, in which the long axes 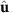 of the particles are aligned in the nematic phase along a common director, whereas for *ν*<0 an oblate _−_ phase is predicted, where the short particle axes 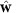 display long-range orientational order. When *v*∼0 a biaxial phase is predicted to be stable, in which both the short and long particle axes show long-range orientational order, provided that other conditions that are strongly shape dependent are also satisfied (for example for rounded board-like particles considered in ref. 9 there is an additional condition of |**u**|/|**w**|≥9).

### Formation of the cholesteric phase

To investigate whether or not the twist in the particle shape is transmitted at a macroscopic level, we perform MC simulations of thousands of TTPs using different initial configurations and boundary conditions, finding consistent results. In this section we present results from MC simulations using standard periodic boundary conditions (PBC) in the NPT ensemble, i.e., at fixed number of particles *N*, pressure *P* and temperature *T*, and we investigate the kinetic pathways for the formation of a prolate cholesteric 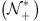 phase.

**Figure 2:**
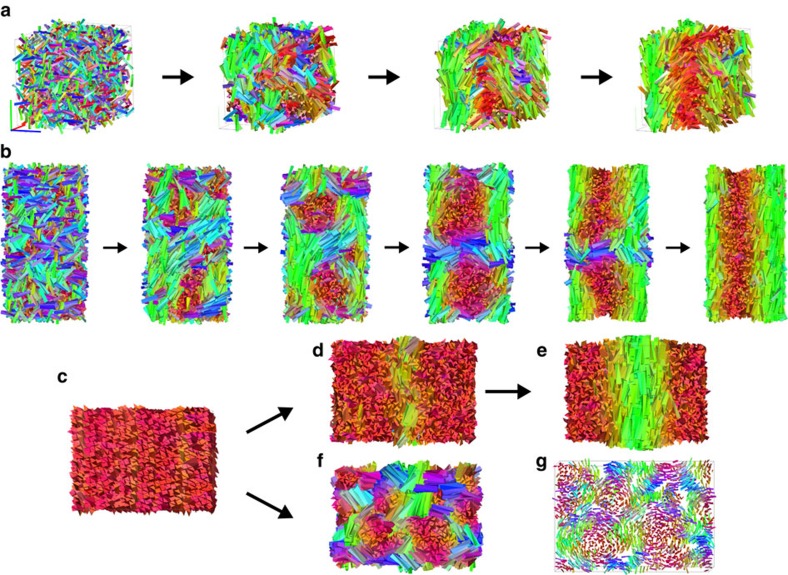
Kinetic pathways for the formation of a prolate cholesteric phase. Typical snapshots obtained from MC simulations in the NPT ensemble under standard periodic boundary conditions are shown. The particles are coloured according to the orientation of their main axis 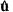. (**a**) Snapshot time series of 2,000 TTP with aspect ratio *h*/*ω*=5, base angle *γ*=1.0 and particle twist angle *α*=0.7, starting from an isotropic state and imposing a pressure *βPω*^3^=1.5 (Supplementary Movie 1). Evolution in time corresponds to an increase of the system packing fraction. The resulting phase is a left-handed cholesteric. (**b**) Snapshot time series of 3,200 TTP with *h*/*ω*=5, *γ*=0.75 and *α*=0.7, starting from an isotropic state and imposing *βPω*^3^=1.9 (Supplementary Movie 3). In this case a spinodal instability is observed: first chiral nematic domains are formed, resembling a blue phase, which slowly merge into a cholesteric defect-free phase. (**c**) Dense aligned state of 2,304 TTP with *h*/*ω*=5, *γ*=0.75 and *α*=0.7 used as initial configuration for expansion runs. (**d**,**e**) At pressure *βPω*^3^=2.1 the twist slowly propagates from the centre of the system (**d**) to the entire system (**e**) and also in this case a cholesteric phase is formed (Supplementary Movie 4). (**f**) At higher pressure (*βPω*^3^=2.9) an instability with respect to nematic orientational fluctuations as well as smectic layering is observed (Supplementary Movie 5), resulting into a metastable state of chiral domains composed of highly aligned particles, which bears close resemblance to a blue phase. See also **g** where the particle size is reduced.

**Figure 3:**
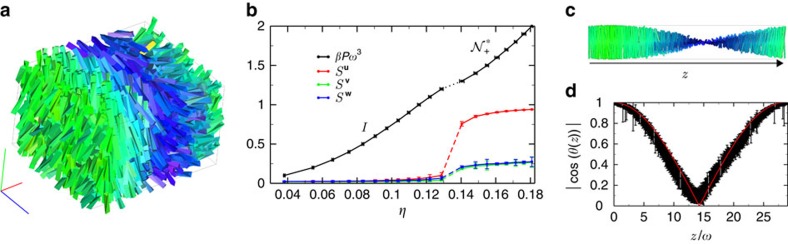
Formation of a prolate cholesteric phase under standard PBC. (**a**) Typical configuration as obtained from simulations of a prolate cholesteric 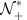 phase of TTPs with aspect ratio *h*/*ω*=5, twist angle *α*=0.7 and base angle *γ*=0.75 using PBC. Particles are coloured according to the orientation of their main axis 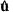. (**b**) Equation of state and nematic order parameters *S* associated to the particle main axis **u**, **v** and **w**, as a function of the packing fraction *η*. Error bars are calculated by averaging 100 independent equilibrated configurations. (**c**) Nematic director profile 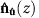, where the orientation of the up-down symmetric rod denotes the direction of the nematic director. The rods are colour coded according to their orientation and the view is in the *xz* plane. (**d**) The twist is quantified by measuring |cos(*θ*(*z*))|, with *θ* the twist angle along the chiral director, i.e., the *z*-direction. The fit used to extract the cholesteric pitch 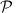 is indicated by the red line, error bars are calculated by averaging 100 independent equilibrated configurations (see text and Methods for more details).

We first consider 2,000 TTPs with a strong particle twist *α*=0.7, and with an aspect ratio *h*/*ω*=5 with an almost equilateral base *γ*=1.0, yielding *v*≃3.25>0. We start from an isotropic phase (*I*) and perform a compression by fixing the pressure *βPω*^3^=1.5 with *β*=1/(*k*_B_*T*) and *k*_B_ Boltzmann's constant. Figure 2a clearly demonstrates the formation of a prolate nematic phase 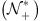 with a spontaneous macroscopic left-handed twist upon increasing the density. The resulting structure has been characterized using appropriate order parameters as shown in Fig. 3 and described in detail in the following section. We note here that the opposite handedness of the cholesteric phase with respect to the particle twist is consistent with theoretical predictions^23^,^24^,^28^,^36^ for chiral particles with a large molecular pitch *p*, in this case *p*/*ω*≃44.8. The phase transformation from *I* to 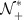 is driven first by nematic fluctuations due to the anisotropy of the overall particle shape, and subsequently, the nematic phase becomes twisted as a result of the finer details of the chiral particle shape. Additionally, we confirm the stability of the 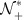 phase by starting from a uniaxial nematic state. We observe that the achiral order is clearly unstable since a twist starts to propagate slowly throughout the whole system as shown in Supplementary Movie 2. Our simulations show that a stable prolate cholesteric phase is found for a large range of twist angles, i.e., 0≲*α*≲*γ*, and base angles 0.55≲*γ*≲*π*/3.

We now investigate in more detail the kinetic pathways leading to 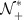 in case of short chiral rods. To this end, we focus on large systems of TTPs with aspect ratio *h*/*ω*=5, an isosceles base with angle *γ*=0.75 and a particle twist angle *α*=0.7, yielding a shape parameter *v*≃1.62>0. First, we determine the pressure *βPω*^3^ as a function of packing fraction *η* as shown in Fig. 3b. From the equation of state, we find that the *I*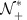 transition occurs at a pressure *βPω*^3^≃1.25, and the transition from a cholesteric phase to a higher ordered one, that we generically denote as chiral smectic (Sm*), takes place at a pressure *βPω*^3^≃2.6 (see Supplementary Fig. 2 for more details). A closer look to the formation of the cholesteric phase reveals that for sufficiently high supersaturation of the isotropic phase (*βPω*^3^=1.9), the transformation proceeds via spinodal decomposition, in analogy with achiral short spherocylinders^18^, see Fig. 2b. We clearly observe that the system is unstable as immediately many small nematic clusters with different orientations are formed throughout the system, which subsequently start to twist. Interestingly, the intermediate phase looks remarkably similar to a blue phase^15^,^33^, but after a long equilibration time the twisted nematic domains start to merge and the system relaxes to a cholesteric phase. Finally, we also study the phase transformation starting from a dense aligned phase. We perform NPT-MC simulations both at a pressure *βPω*^3^=2.1 corresponding to a stable 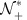 phase, and at *βPω*^3^=2.9, where the Sm* phase is expected to be stable. For *βPω*^3^=2.1, we indeed find that the director field immediately starts to twist in the aligned phase of TTPs, resulting into a cholesteric phase as shown in Fig. 2c,d. On the other hand, at *βPω*^3^=2.9, the system is unstable with respect to both nematic orientational fluctuations as well as smectic layering fluctuations as seen in Fig. 2e, and again the final structure bears close resemblance to a blue phase, which corresponds to a probably metastable (kinetically arrested) phase as the Sm* is expected to be the stable state. However, this suggests an intriguing competition between packing and chirality at high pressures that will be further investigated in future studies.

### Equilibrium cholesteric pitch

We now turn our attention to the helical structure of the cholesteric phase. To this end, we measure the equilibrium cholesteric pitch 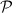, a parameter that quantifies the macroscopic chirality, and study how it is affected by boundary conditions and finite-size effects. To this end, we analyse the spatial dependence of the nematic director by dividing the system into slabs^32^,^33^ along the axis of the macroscopic twist, also called chiral director, which is oriented along the *z* axis. For each slab, we compute the local nematic director 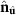 and nematic order parameter *S*^**u**^ by diagonalizing the tensor 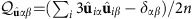, where 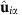 denotes the *α*=*x*, *y*, *z*-component of the long particle axis 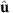 of particle *i* with *i*=1, ..., *n*, and *n* the number of particles in the slab. *S*=*S*^**u**^ is the largest eigenvalue of 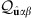 with 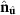 the corresponding eigenvector. The same procedure is repeated to obtain *S*^**v**^ and *S*^**w**^, which are the largest eigenvalues of 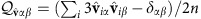 and 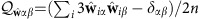 with 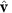 and 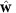 the medium and short particle axis, respectively. Again, 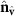 and 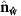 are the associated eigenvectors. We show an example of such a nematic director profile 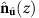 in Fig. 3c, where the orientation of an up-down symmetric rod denotes the direction of the nematic director along the chiral director (*z* axis). Note that we assume 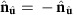. The corresponding nematic order parameter *S*^**u**^ is displayed in Fig. 3b together with *S*^**v**^ and *S*^**w**^. The twist is quantified by averaging 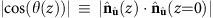 over hundreds of independent configurations and by performing a one-parameter fit using 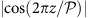, we extract the cholesteric pitch 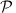, shown with red lines in Fig. 3d. In addition, we compute orientational pair-correlation functions along the chiral director, as introduced in ref. 31, to confirm the helical structure and the sense of the macroscopic twist (see Methods and Supplementary Fig. 3 for more details).

It is important to note that in the case of PBC the nematic director should be the same at the edges of the simulation box, i.e., cos(*θ*(*z*=*L*_*z*_))=1 in Fig. 3d with *L*_*z*_ the box length in the *z*-direction. As a consequence, the cholesteric pitch 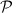 must be commensurate with *L*_*z*_, i.e., *L*_*z*_ should be at least 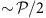 to observe a twist in the nematic phase^31^. By allowing the box shape to relax, either by performing NPT simulations or NVT simulations using a variable box shape, we expect the accuracy of the equilibrium pitch measurement to improve, but by repeating our simulations for different system sizes, we still observed a dependence on the initial box size (Supplementary Fig. 4a).

**Figure 4:**
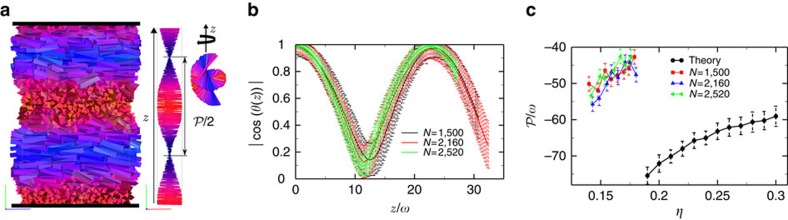
Prolate cholesteric phase embedded between two hard walls. (**a**) Typical configuration of a left-handed prolate 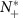 cholesteric as obtained from NVT-MC simulations on systems of TTPs with aspect ratio *h*/*ω*=5, twist angle *α*=0.7 and base angle *γ*=0.75 confined between two planar hard walls (in the *z*-direction). The particles are coloured according to the orientation of their main axis 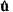. A view in the *xz* plane and a bird's eye view of the nematic director profile 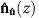 as a function of *z*, i.e., along the chiral director, where the orientation of the up-down symmetric rod denotes the direction of the nematic director. The rods are colour coded according to their orientation. (**b**) Nematic director profiles |cos(*θ*(*z*))| obtained by simulations using different numbers of particles *N* and varying box sizes, but at fixed packing fraction *η*≃0.16. Error bars are calculated by averaging 100 independent equilibrated configurations. (**c**) Cholesteric pitch 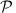 versus packing fraction *η* as obtained from simulations (using hard walls) along with predictions from a second-virial density functional theory^28^,^36^. Simulation error bars are estimated from the fits whereas theoretical error bars are obtained by averaging results from 10 independent calculations based on 10^10^ pairs of particles.

To circumvent the commensurability problems with pitch and box size, we embed the system between two planar hard walls in such a way that the nematic director can freely choose its orientation at both walls, and we perform simulations in the NVT ensemble. As can be observed from Fig. 4a,b, the nematic director profile is indeed not commensurate anymore with *L*_*z*_ thereby allowing for a full relaxation of the macroscopic chiral twist. Since we simulate sufficiently large system sizes at state points that are well inside the stable region of the cholesteric phase, we expect that surface effects, such as pronounced layering or increased biaxiality^34^,^39^, should be negligible. We indeed observe that the walls only affect the structure at distances smaller than ∼1 particle diameter from the wall (Supplementary Fig. 5). To support this, we determine the equilibrium pitch 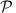 using different system sizes (different number of particles and different box dimensions), but at fixed packing fraction *η*. Panels b and c of Fig. 4 show indeed consistent results for the nematic director profile as well as for the value of 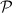. We thus regard this method to be the most convenient and reliable way for calculating the equilibrium pitch 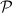, in analogy with the conclusions of ref. 32.

Finally, we also perform simulations using twisted boundary conditions (TBC)^40^. We find good equilibration of our cholesteric phases of TTPs, as evidenced in Supplementary Fig. 4(b) by the much smaller error bars on the nematic director profile and the difference of *π*/2 in the *θ* angle at the edges of the box as imposed by the TBC. However, the use of TBC may result into an over- or undertwisted cholesteric phase, and only by measuring the pressure tensor, which is unfortunately not straightforward for hard particles, it would be possible to extract the equilibrium value of 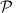 (ref. 41). This procedure is based on a quadratic approximation around the free-energy minimum^41^ and TTPs will be a suitable system to test this approach.

### Comparison with second virial theory

**Figure 5:**
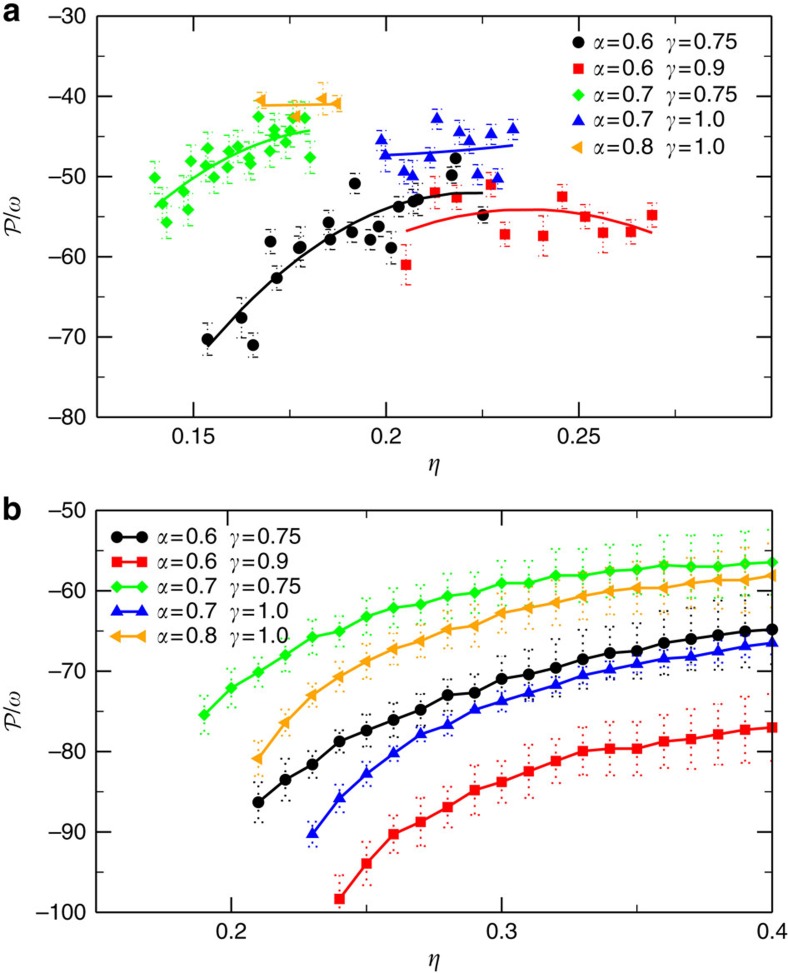
Comparison between simulations and theoretical predictions. (**a**) Cholesteric pitch 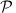 versus packing fraction *η* as obtained by MC simulations (using hard walls) for TTPs with varying twist angles *α* and base angle *γ* as labelled. Lines are polynomial fits used as guides to the eye. Error bars are estimated from the fits. (**b**) Theoretical predictions for the same particle models obtained by second-virial DFT^28^,^36^. Error bars are obtained by averaging results from 10 independent calculations based on 10^10^ pairs of particles. An increase in the twist angle *α* while keeping fixed the base angle *γ* corresponds to a shorter cholesteric pitch 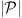. Analogously, keeping fixed *α* and decreasing *γ* enhance the particle chirality and yields a shorter 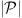. However, this effect seems to be overestimated by theory resulting in a slightly different chiral ranking for the models considered here. In Supplementary Fig. 6a the same results are shown by using the nematic order parameter instead of the packing fraction and similar conclusions are drawn.

The availability of cholesteric phases obtained from particle-based simulations provides a new testing ground for the theoretical framework describing the chiral organization in liquid crystals. We apply the recently developed second-virial density functional theory (DFT)^28^,^36^ to our system of TTPs and calculate the density dependence of 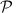. Our DFT is an extension of Onsager's theory^1^ corrected with a Parsons–Lee prefactor to deal with the finite size of the particles^42^,^43^. It represents an advancement over Straley's approach^23^ as it does not consider the chirality in a perturbative way^37^ and it is combined with a MC integration to make it suitable for a wide range of particle models^36^. A detailed description can be found in refs 28, 36. In Fig. 4c we present our results as obtained from simulations along with the DFT predictions. We plot 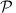 as a function of *η* in the range where the cholesteric phase is stable. We observe that the theory correctly captures the sense of twist, the magnitude and the trend of 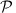 as a function of *η*. In addition, we study the effect of particle shape on the cholesteric pitch 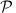. In Fig. 5a, we present simulation results for the pitch 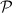 as a function of *η* for varying twist angle *α* and base angle *γ*. Comparing the results for *α*=0.6 (red curve) with *α*=0.7 (green) at fixed *γ*=0.75, or *α*=0.7 (blue) with *α*=0.8 (yellow) at *γ*=1.0, reveals that 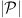 decreases upon increasing *α*, i.e., increasing the microscopic chirality of the particle, as expected. Analogously, by decreasing the base angle *γ*, the surface associated with the longer side of the base gets larger, which effectively increases the particle chirality, thereby yielding a smaller pitch 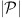. This trend can be appreciated by comparing the results for *γ*=1.0 (blue) with *γ*=0.75 (green) at fixed *α*=0.7 or *γ*=0.9 (red) with *γ*=0.75 (black) at fixed *α*=0.6. Despite an overall little underestimation of the macroscopic twist, Fig. 5b shows that all these trends are well-captured by our DFT calculations: increasing the particle chirality, by either twisting the particle more (increasing *α*) or increasing the particle biaxiality (by decreasing *γ*), results indeed in a smaller cholesteric pitch 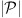. However, we notice that the effect of decreasing *γ* on particle chirality is overestimated by the DFT with respect to the results obtained from simulation of the many-particle system. Nevertheless, the DFT can be used as a reliable and quick tool for predicting the macroscopic chiral behaviour from the microscopic chiral particle properties. We therefore use our theory to study the effect of TTPs with multiple twists on the cholesteric pitch . Our DFT calculations as shown in Supplementary Fig. 6(b) reveal that upon decreasing the microscopic pitch length *p*/*ω*, the sense of the macroscopic twist changes from opposite to same handedness with in between a regime where a twist inversion occurs with packing fraction, which is analogous to previous results on hard helices^28^,^36^. In the conclusions we will discuss possible improvements for the theoretical framework.

### Right-handed oblate cholesteric phase

**Figure 6:**
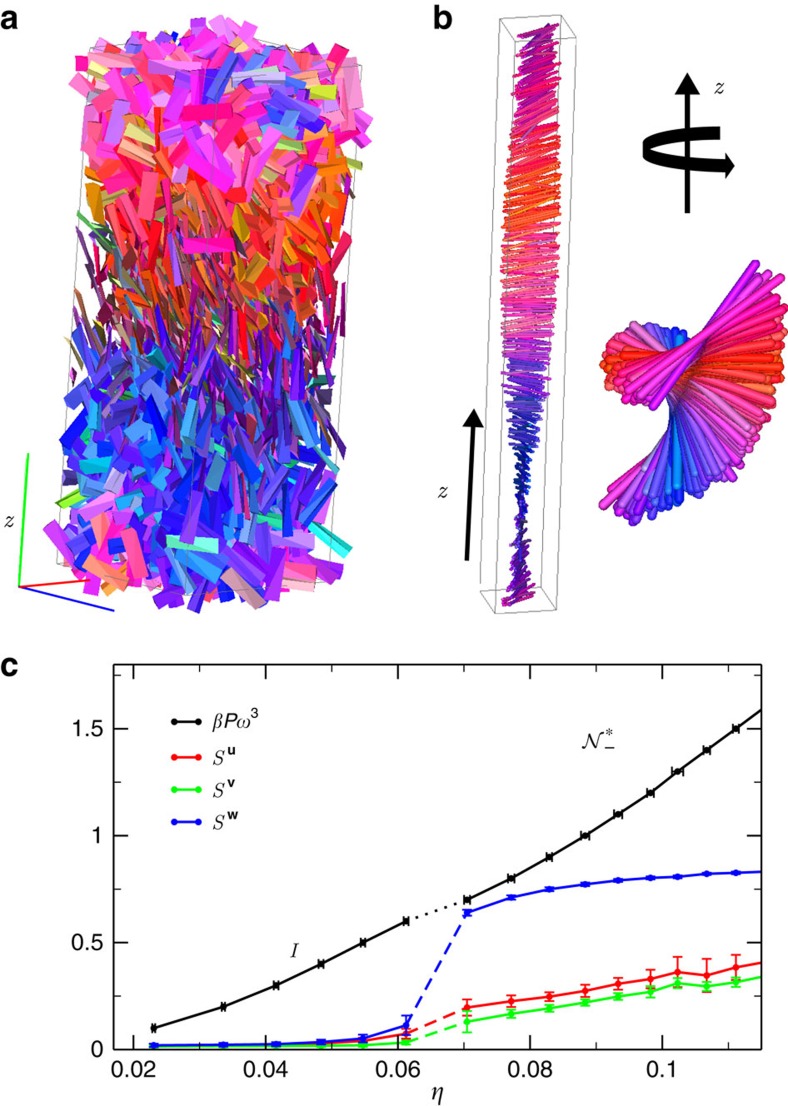
Formation of an oblate cholesteric phase under standard PBC. (**a**) Oblate cholesteric 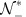 phase of TTPs with *h*/*ω*=5, *α*=0.4 and *γ*=0.4 as obtained from NPT-MC simulations (*βPω*^3^=1.0) using PBC. Particles are coloured according to the orientation of their short particle axes 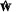. (**b**) Two bird's eye views of the nematic director profile 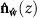 as a function of *z*, where the orientation of the up-down symmetric rod denotes the direction of the nematic director. The rods are color-coded according to their orientation, and display a right-handed twist. (**c**) Equation of state and nematic order parameters *S*^**u**^, *S*^**v**^, *S*^**w**^ as a function of packing fraction *η*, confirming that the transition is specular to the isotropic-prolate cholesteric phase transition (cfr. Fig. 3b). Error bars are calculated by averaging 100 independent equilibrated configurations.

We further modify the particle shape by decreasing the base angle *γ* while keeping the aspect ratio *h*/*ω*=5 fixed. In this way, we construct TTPs with shape parameter *ν*<0, which should stabilize an oblate nematic phase. Indeed, our simulations reveal the formation of an oblate nematic phase 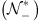 with a helical chiral arrangement of the local nematic director field corresponding to the short particle axis 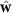 as exemplarily shown in Fig. 6 for TTPs with *h*/*ω*=5, *γ*=0.4, *α*=0.4, yielding *v*≃−1.41. We observe that the orientation of the long particle axis 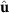 is isotropic whereas the nematic director 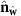 associated to the short axis 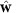 displays the expected helical structure. Surprisingly, the macroscopic twist is now right-handed in contrast with the left-handed twist as observed for the prolate cholesteric phase of the same particle model but with a different *α* and *γ* (Fig. 3), which seems to be counter-intuitive. However, this can be explained as follows. Despite the fact that the twist angle *α*>0, meaning that the particle is twisted in a right-handed fashion along the long particle axis 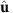, it also corresponds to a left-handed twist in the short particle axis 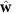. As only the short particle axes show orientational order in an oblate nematic phase, and the particles are weakly chiral, we expect a macroscopic twist that is opposite to that of the short axis, i.e., a right-handed macroscopic twist, as indeed observed in our simulations. Supplementary Movie 6 (see also equation of state and order parameters in Fig. 6c) shows that the *I*–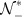 phase transformation is specular to that of the prolate cholesteric phase.

### Towards design rules for hard chiral particles

**Figure 7:**
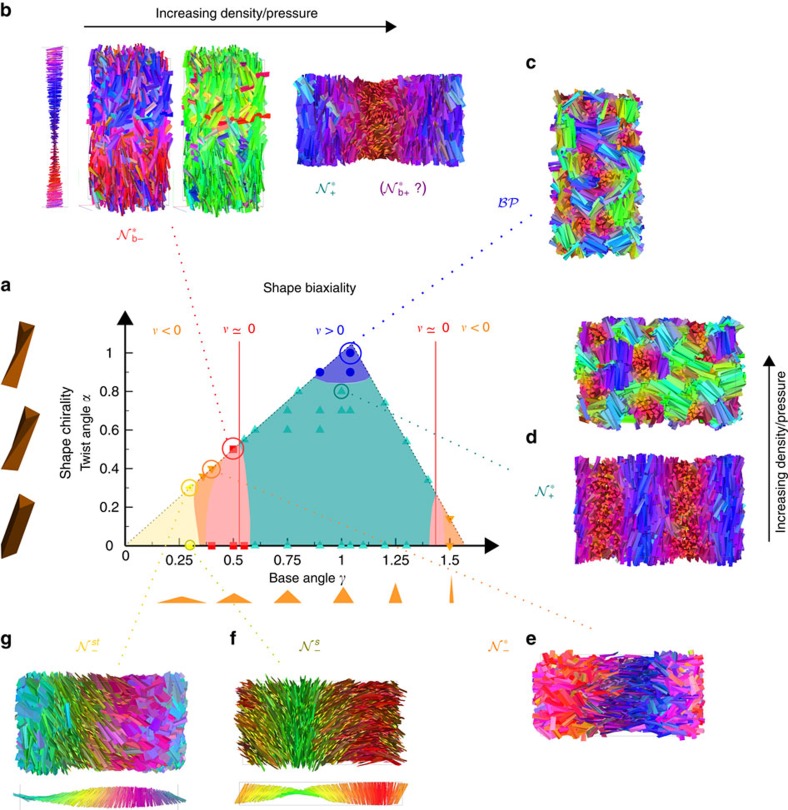
Chiral nematic behaviour of hard twisted triangular prisms. (**a**) Nematic phase behaviour for twisted triangular prisms of height *h*/*ω*=5 in the twist angle *α* - base angle *γ* representation obtained by computer simulations compressing from an isotropic phase. Regions are coloured according to the type of nematic phase that is found stable (white corresponds to unphysical parameters), see text for a detailed description. (**b**) Left: 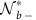 phase with nematic director profile 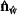, a configuration with particles coloured according to 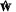 and according to 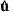. Right: 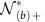 phase with configuration of nematic phase at higher pressure, and particles coloured according to 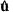. (**c**) 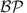 phase, particles coloured according to 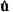. (**d**) 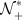 and 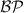 (at higher pressure) with particles coloured according to 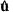. (**e**) 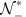 with particles coloured according to 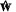. (**f**) 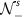 with particles coloured according to 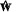 and corresponding nematic director profile of 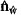. (**g**) Snapshot of 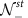 with particles coloured according to 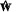 and corresponding profile of 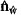.

Finally, we perform many simulations on systems of TTPs with height *h*/*ω*=5, and study the nematic phase behaviour for different values of the base angle *γ* and twist angle *α*. In Fig. 7a we show our results in the *γ*–*α* plane. As reference, we also report results for *α*=0, corresponding to achiral triangular prisms. We observe that by tuning the shape chirality, i.e., changing *α*, and the shape biaxiality, i.e., changing *γ*, several phases can be formed upon compression from an isotropic phase (under PBC). In particular, a prolate (calamitic) chiral nematic 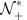 phase is observed when *ν*>0 (light-blue region), as already shown above. However, the nematic state can also change upon increasing the packing fraction. For instance, TTPs with a relatively high twist *α* (and *ν*>0) undergo a transition from a 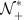 phase to a phase that is reminiscent of the blue phase 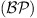, as shown in Fig. 7d. Indeed, the 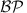 exhibits no long-range orientational order as evidenced by a vanishing nematic order parameter, but the particles are strongly aligned with respect to their neighbouring particles, which poses the question whether this 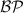 may actually be a twisted grain boundaries phase with very small smectic-like domains. Interestingly, for TTPs with the strongest twist *α* (dark-blue region) the 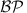 is observed directly from the isotropic phase without an intervening nematic phase. The 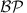 seems to be surprisingly stable as even upon increasing the pressure further this phase remains. On the other hand, for *ν* <0 an oblate (discotic) 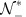 chiral nematic phase is formed, in which the short particle axes display long-range orientational order (orange and yellow regions). It is worth noting that there are two regions where the 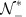 phase is stable, i.e., for sufficiently small or sufficiently large base angle *γ*. However, in the case that the triangular base is extremely flat (when *γ* is very small, and thus also *ν*) an additional splay deformation is formed in the nematic director field 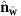 corresponding to the short particle axis 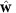, therefore forming a twist-splay nematic 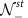 (yellow region), as shown in Fig. 7g. Such a deformation is not generic for chiral particles but seems to be specific to triangular prisms, which is corroborated by the fact that for the achiral particle with same *γ*, an achiral nematic phase is observed with also a splay deformation, i.e., a 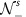 phase, as shown in Fig. 7f. Further studies on the stability of this deformation are required but could be interpreted as another modulated nematic phase.

Finally, we focus on the region where *v*≃0 (red region). Interestingly, we clearly observe the formation of biaxial nematic phases for both achiral and chiral triangular prisms in which both the short 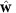 and long 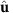 particle axes show long-range orientational order. Surprisingly, we find that in the case of twisted triangular prisms, only one of the nematic directors show a chiral helical arrangement. To be more specific, we find at low densities that a biaxial nematic phase is formed in which only the nematic director corresponding to the short particle axis show a helical arrangement, resulting in a 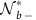 phase as shown in Fig. 7b. Upon increasing the density, this biaxial chiral nematic 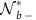 phase transforms into a (biaxial) chiral nematic 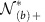 phase with a helical arrangement of the nematic director associated to the long particle axes. As the orientational order of the short particle axes is very weak, we could not identify if the chiral nematic phase is uniaxial 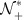 or biaxial 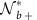. A transition from 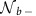 to 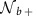 is consistent with the simulations on hard board-like particles^9^, where the achiral biaxial phases were characterized by the particle axis with the highest orientational order. It is tempting to speculate that indeed only the nematic director of the particle axis that shows the strongest alignment becomes chiral in a biaxial nematic phase, as it seems unlikely that both director fields twist simultaneously and remain space-filling. However, it is impossible to rule out such a scenario of double twist as the cholesteric pitch of one or both director fields can easily exceed the system sizes that are at present accessible in simulations.

In summary, we map out the nematic phase behaviour in the shape biaxiality-shape chirality parameter space and we show how the observed nematic phases can be rationalized: (i) The shape parameter *ν* can be used to predict which of the two particle axes exhibits long-range orientational order, resulting in a discotic 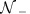 nematic phase for *ν*<0 or a calamitic 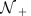 nematic phase when *ν*>0. For *v*∼0, a biaxial phase may be present, provided that other conditions that depend on the exact particle shape are satisfied. This was already demonstrated in a simulation study on hard board-like particles^9^, where the particle aspect ratio needs to be larger than a threshold value to observe a biaxial nematic phase. (ii) Furthermore, we find that in the case that the triangular base is extremely flat, the nematic phase displays a splay deformation for both chiral as well as achiral particles. (iii) In addition, we find that for highly twisted particles or at high pressures blue phases can be stabilized. (iv) Our results also reveal that the coupling between particle biaxiality and chirality is highly non-trivial as the prolate nematic phases are left handed, whereas the oblate nematic phases are right handed using the same right-handed particle model. We have to remark that to assess the thermodynamic stability and the corresponding phase boundaries of all of these phases, accurate free-energy techniques to apply in computer simulations and an extended density functional theory that takes in account all of these effects at the same time, are needed but not yet developed.

## Discussion

In conclusion, we show by computer simulations of twisted triangular prisms that entropy alone can stabilize both oblate (discotic) and prolate (calamitic) chiral nematic phases. Our results showcase once more that attributing uniquely a value to the microscopic chirality is not trivial. In this simple model we need to combine the value of the twist angle *α*, shape parameter *ν* and the microscopic pitch *p* to predict the sense of twist. A more complicated competition between biaxiality and chirality is expected when *v*∼0 and a biaxial nematic phase should occur. Our simulations reveal the formation of biaxial nematic phases of twisted triangular prisms in which both the short 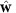 and long 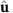 particle axes show long-range orientational order, but only the nematic director corresponding to the strongest alignment show a chiral helical arrangement. It is interesting to investigate if this scenario is generic or if it is possible that both nematic directors exhibit a chiral twist, which seems unlikely as a double twist cannot be system spanning. Twisted polyhedra will be useful models to address this and other fundamental questions. For example, preliminary simulations on mixtures of particles with different handedness show that racemic mixtures form achiral nematic phases, as expected from theory^15^, indicating that chirality alone is not enough to drive phase separation in systems of hard particles and size asymmetry is required. By considering also depletant particles it will be possible to gain novel insights in experiments where entropy, chirality and depletion are the dominant forces^44^,^45^.

In addition, our simulations show qualitative agreement with theoretical predictions from an Onsager-like DFT, thereby providing confidence that the theory yields reliable results and can thus serve as a guide for future studies. For example, to study nucleation and growth of cholesteric phases, addressing questions like how the chirality changes the shape of the nematic nucleus, longer particles are needed^18^ for which 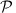 is expected to be larger and therefore a careful choice of the shape is essential. However, it is also evident that the Parsons–Lee correction does not rescale the packing fraction of twisted polyhedra accurately enough and overestimates the *I*– transition. This may be remedied by a better rescaling factor than the Parsons–Lee correction, or by a more accurate microscopic theory such as fundamental measure theory. The DFT also overestimates 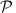 compared with simulations, i.e., it underestimates the macroscopic chiral twist of a phase. A similar conclusion was also drawn in previous work on attractive chiral spherocylinders^32^. These issues need further investigations.

Finally, recent advancements in chemical synthesis of nanocrystals with polyhedral shape^46^ and the use of polarized light to introduce chirality in the shape^47^, brings optimism on the possibility of achieving control over more and more particle features, including chirality, at the microscopic level. It is worth mentioning that the twisted prisms resemble the twisted nanoribbons as synthesized in refs 47, 48. We hope that our study motivates further theoretical efforts in the directions of chiral particles. Computer simulations of hard particles will be helpful in the shape design of future building blocks. Moreover, our results, together with the exhaustive literature on hard particles, pose the intriguing question if any thermodynamic phase featuring long-range order can be stabilized by entropy alone. Several shape features have been identified as responsible for the stabilization of different thermodynamic phases and therefore design rules for novel building blocks start to take shape.

## Methods

### Computer simulations

For TTP with *γ*≤*π*/3, |**v**|=*π* cos*γ*/(1+cos*γ*) and |**w**|=*π* sin*γ*/(2+2 cos*γ*), such that the shape parameter *ν*=*h*(1+cos *γ*)/*π*cos *γ*−2/tan *γ*, where *h*=|**u**| is the height of the particle. The volume of the particle is calculated by using standard formulas for orientable polyhedra that requires the knowledge of face normals and vertices positions. Overlaps between particles are detected by checking intersections between triangular faces using the RAPID library (http://gamma.cs.unc.edu/OBB/). Thousands of particles are simulated using standard MC simulation methods in either the NVT or NPT ensemble. In the former, MC moves consist in either single-particle translation or rotation whereas in the latter also volume-change moves (both isotropic and anisotropic scaling) are employed. Several millions of MC steps are performed both in the equilibration and production runs, where one MC step is defined as *N* moves, with *N* the number of particles. Different boundary conditions and initial configurations are used, as specified in the text. In the case of hard walls (located at *z*=0 and *z*=*L*_*z*_), the overlap detection between particles and walls is performed by checking if any vertices of the polyhedra have coordinates *z*<0 or *z*>*L*_*z*_. For the implementation of twisted boundary conditions we refer to ref. 40. To obtain the equilibrium equation of state we combined results obtained by starting from an isotropic configuration, from a dilute lattice and from a dense aligned lattice (constructed by first obtaining the closest packing of a few particles in an orthogonal cuboidal box). States equilibrated at close pressures are also used as initial configurations to avoid kinetic traps. To determine the transitions between different thermodynamic phases, we have calculated several order parameters in both the NPT and NVT ensembles. Using equilibrated configurations we also set up long (>6 × 10^6^ MC steps) NVT simulations to accurately measure the cholesteric pitch 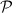. After dividing the system in slabs, we compute the local nematic directors 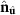, 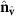 and 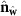, and nematic order parameters *S*^**u**^, *S*^**v**^ and *S*^**w**^ as described in the main text, and where we neglect the polar nature of the particle as we assume up-down symmetry 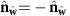. However, we checked that it did not affect our results. For each configuration we calculate 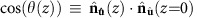 and we bypass the up-down symmetry by taking the absolute value. After averaging hundreds of such profiles, we perform a one-parameter fit using 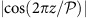 to extract the cholesteric pitch 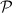. Although our procedure removes the (small) intrisinc drift of the system occuring over different configurations, the statistical error on 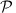 is still on the order of several *ω* as shown in Fig. 3c. Averaging the profiles *S*(*z*), the bulk values are obtained for each state points. The cholesteric helical structure and the sense of the twist are further confirmed by orientational pair-correlation functions as introduced in ref. 31 and reported in Supplementary Figs 3 and 5.

### Classical density functional theory

The simulation results are compared with those obtained using a second-virial classical density functional theory^28^,^36^. The input of such theory is the pitch-dependent Legendre-expanded excluded-volume between two particles with orientation 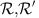 separated by a distance **r**:


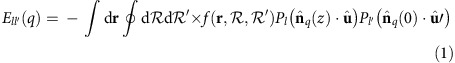


with *P*_*l*_ the normalized Legendre polynomial of grade *l*=0,2,...,20 (only even coefficients are considered), 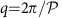 the chiral wave vector, 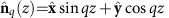 the nematic director profile and *f* the Mayer function that assumes a value −1 if particles overlap and 0 otherwise. The coefficients *E*_*ll*′_(*q*) are calculated using a MC integration scheme. Once these coefficients are calculated, the orientation distribution function 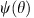, with *θ* the polar angle with respect to the local nematic director, is obtained by minimizing a Parsons–Lee–Onsager-like free-energy functional^1^,^42^,^43^ yielding the following equation:


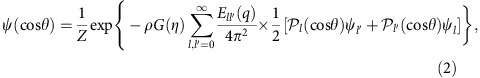


with *ρ* the number density, *G*(*η*) the Parsons–Lee correction, 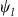 the expansion coefficients of 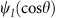 and *Z* the normalization factor. Finally, the equilibrium pitch 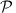 is obtained by inserting back 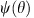 into the functional and identifying the minimum of the free energy.

## Additional information

**How to cite this article:** Dussi, S. & Dijkstra, M. Entropy-driven formation of chiral nematic phases by computer simulations. *Nat. Commun.* 7:11175 doi: 10.1038/ncomms11175 (2016).

## Supplementary Material

Supplementary Information Supplementary Figures 1-6 and Supplementary References

Supplementary Movie 1 Formation of a prolate cholesteric phase from an isotropic phase. MC simulation of N = 2000 TTP with aspect ratio *h*/ω = 5, base angle γ = 1.0, and particle twist angle α = 0.7 using PBC (around 2 × 10^6^ MC steps are shown), starting from an isotropic state and imposing a pressure *βP ω*^3^ = 1.5.

Supplementary Movie 2 Instability with respect to a uniaxial nematic phase. MC simulation of N = 2000 TTP with aspect ratio *h/ω* = 5, base angle γ = 1.0, and particle twist angle α = 0.7 using PBC (around 2 × 10^6^ MC steps are shown), starting from a uniaxial nematic state and imposing a pressure *βP ω*^3^ = 1.5. A prolate cholesteric phase is formed.

Supplementary Movie 3 Spinodal decomposition for chiral short rods. MC simulation of N = 3200 TTP with aspect ratio *h/ω* = 5, base angle γ = 0.75, and particle twist angle α = 0.7 using PBC (around 6 × 10^6^ MC steps are shown), starting from an isotropic state and imposing a pressure *βP ω*^3^ = 1.9. Few chiral nematic domains are formed with different twist orientation (spinodal decomposition), which successively anneal into a defect-free cholesteric phase.

Supplementary Movie 4 Formation of a prolate cholesteric phase from a dense aligned state. MC simulation of N = 2304 TTP with aspect ratio *h/ω* = 5, base angle γ = 0.75, and particle twist angle α = 0.7 using PBC and imposing a pressure *βP ω*^3^ = 2.1.

Supplementary Movie 5 Kinetically-trapped blue-phase-like state. MC simulation of N = 2304 TTP with aspect ratio *h/ω* = 5, base angle γ = 0.75, and particle twist angle α = 0.7 using PBC (around 4 × 10^6^ MC steps are shown), starting from a dense aligned state and imposing a pressure *βP ω*^3^ = 2.9. Instability with respect to nematic orientational fluctuations as well as smectic layering fluctuations is observed, resulting into a kinetically arrested state of chiral domains composed of highly aligned particles, which resembles a blue phase.

Supplementary Movie 6 Formation of an oblate cholesteric phase from an isotropic phase. MC simulation of N = 2400 TTP with aspect ratio *h*/ω = 5, base angle γ = 0.4, and particle twist angle α = 0.4 using PBC, starting from an isotropic state and imposing a pressure *βP ω*^3^ = 1.0. An oblate right-handed cholesteric phase is formed.
