# Supplementary material for: Entropy-driven formation of chiral nematic phases by computer simulations
Source: Nat Commun. 2016 Apr 12;7:11175. doi: 10.1038/ncomms11175 (PMC4832067; doi:10.1038/ncomms11175)
Supplement: Supplementary Information — Supplementary Figures 1-6 and Supplementary References [file ncomms11175-s1.pdf]

SUPPLEMENTARY INFORMATION

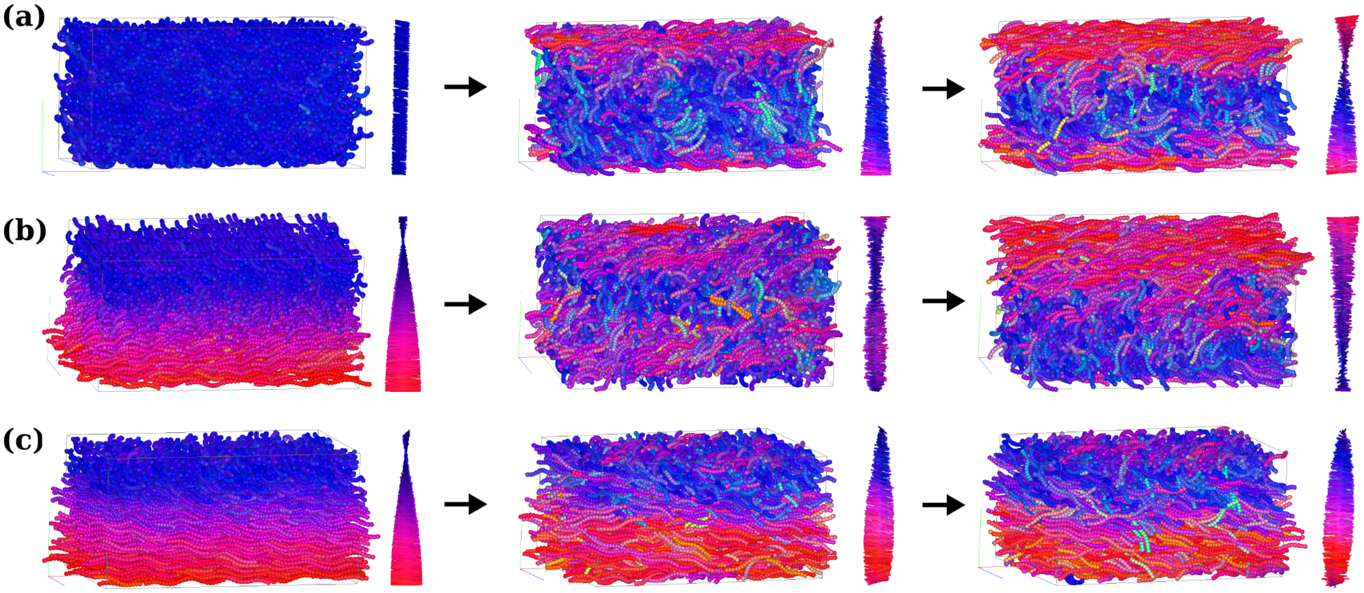

Supplementary Figure 1. **Cholesteric phase of hard helices by computer simulations.** Simulation snapshots of cholesterics of hard helices with $p = 8\sigma$, $r = 0.4\sigma$, $L_c = 10\sigma$, obtained by NVT-Monte Carlo simulations of 2100 particles at a packing fraction $\eta \simeq 0.319$ and confined between two smooth hard walls. Particles are colored according to the orientation of their main axes and the corresponding nematic director profile along the direction perpendicular to the two walls is depicted by the orientation of the rod-like segments. (a) Time-series of configurations starting from an achiral state taken at time zero, after $4 * 10^6$ MC steps and after $9 * 10^6$ MC steps. The system spontaneously twists in a left-handed cholesteric. (b) Starting from a right-handed configuration, snapshots taken at time zero, after $3 * 10^6$ MC steps and after $6 * 10^6$ MC steps. The system twists back. (c) Starting from a left-handed initial configuration, snapshots taken at at time zero, after $3 * 10^6$ MC steps and after $6 * 10^6$ MC steps. The left-handed twist is preserved but the magnitude of the cholesteric pitch changes upon system relaxation.

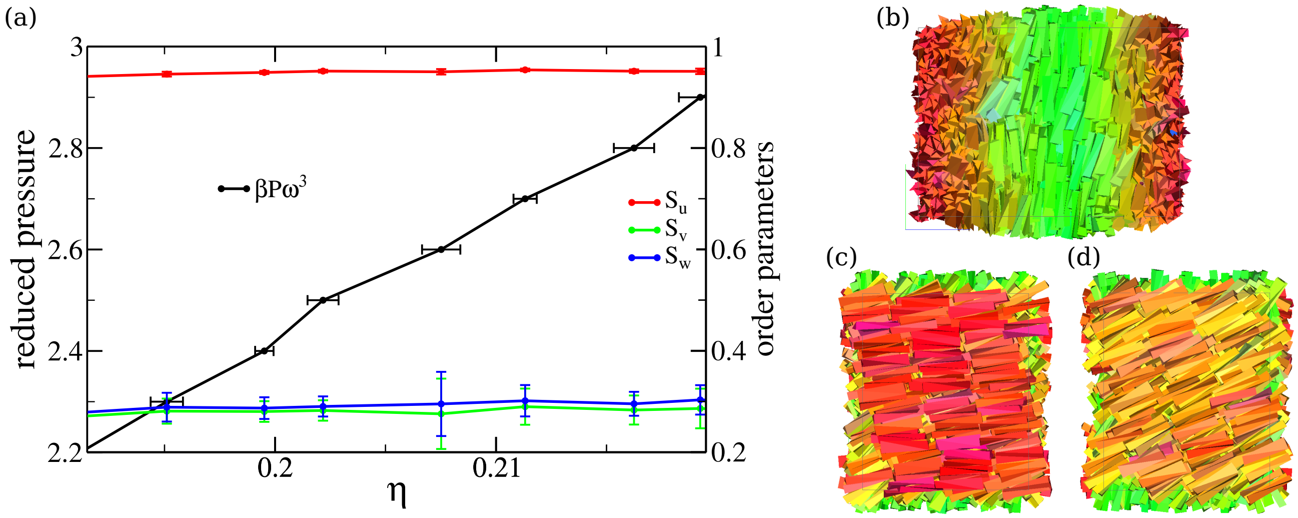

Supplementary Figure 2. **Onset of particle layering.** (a) The pressure $\beta P\omega^3$ (left y-axis) and nematic order parameters S (right y-axis) as a function of packing fraction η as obtained by MC simulations in the NPT ensemble with PBC for TTPs with aspect ratio $h/\omega = 5$, base angle $\gamma = 0.75$, and particle twist angle $\alpha = 0.7$. Snapshots in (b)-(c)-(d) are taken from a typical configuration at $\beta P\omega^3 = 2.9$. At this pressure the helical structure is still mantained (side view in panel (b)) but in the direction perpendicular to the chiral director the particles form smectic layers (panels (c) and (d) show cuts through the sample), corresponding to a chiral smectic phase generically denoted as Sm^*. Due to the small number of layers and particles in each slabs of the system the use of ordinary translational smectic order parameter does not locate accurately the transition from the cholesteric to Sm^*, that is therefore estimated by visual inspection of the configurations. The exact identification of the phase, that is more likely a twisted grain boundary chiral smectic A, and its stability, are beyond the scope of the current study.

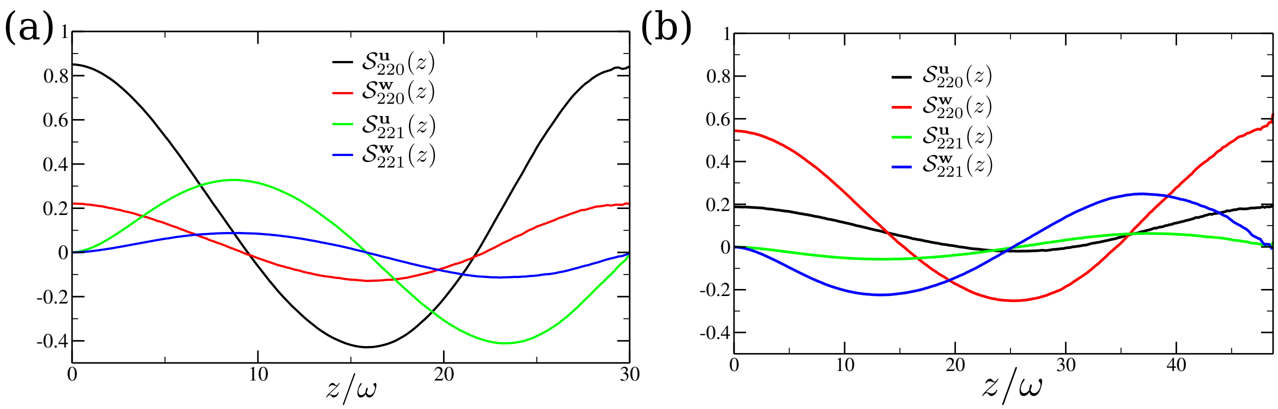

Supplementary Figure 3. **Orientational pair-correlation functions.** Orientational pair-correlation functions [1] calculated along the chiral director (parallel to the z-axis) in case of TTPs with $h/\omega = 5$ and (a) $\gamma = 0.75$, $\alpha = 0.7$ forming a prolate cholesteric and (b) $\gamma = 0.4$, $\alpha = 0.4$ forming an oblate cholesteric phase. The systems are simulated by using PBC. $\mathcal{S}^{\mathbf{u}}_{220}(z) = \langle 3/2(\hat{\mathbf{u}}_i \cdot \hat{\mathbf{u}}_j)^2 - 1/2\rangle$ describes the nematic order between two particles i and j separated by distance $z = z_{ij}$, with $\langle . \rangle$ indicating an average over all the particles and different configurations. The distance between the maximum and the minimum corresponds to half cholesteric pitch. $\mathcal{S}^{\mathbf{u}}_{221}(z) = \langle [(\hat{\mathbf{u}}_i \times \hat{\mathbf{u}}_j) \cdot \hat{\mathbf{z}}_{ij}](\hat{\mathbf{u}}_i \cdot \hat{\mathbf{u}}_j)\rangle$ describes the chiral organization between the two particles i and j along the z-axis. Analogously, the functions $\mathcal{S}^{\mathbf{w}}_{220}(z)$ and $\mathcal{S}^{\mathbf{w}}_{221}(z)$ refer to the short axis $\hat{\mathbf{w}}$. The sinusoidal oscillations typical of the cholesteric helical structures are evident both in the prolate (a) and oblate (b) case, with the long and short, respectively, axes featuring an higher degree of orientational order. For the prolate phase (a) the macroscopic twist is left-handed whereas for the oblate (b) is right-handed as confirmed by the opposite trends of the functions $\mathcal{S}_{221}(z)$.

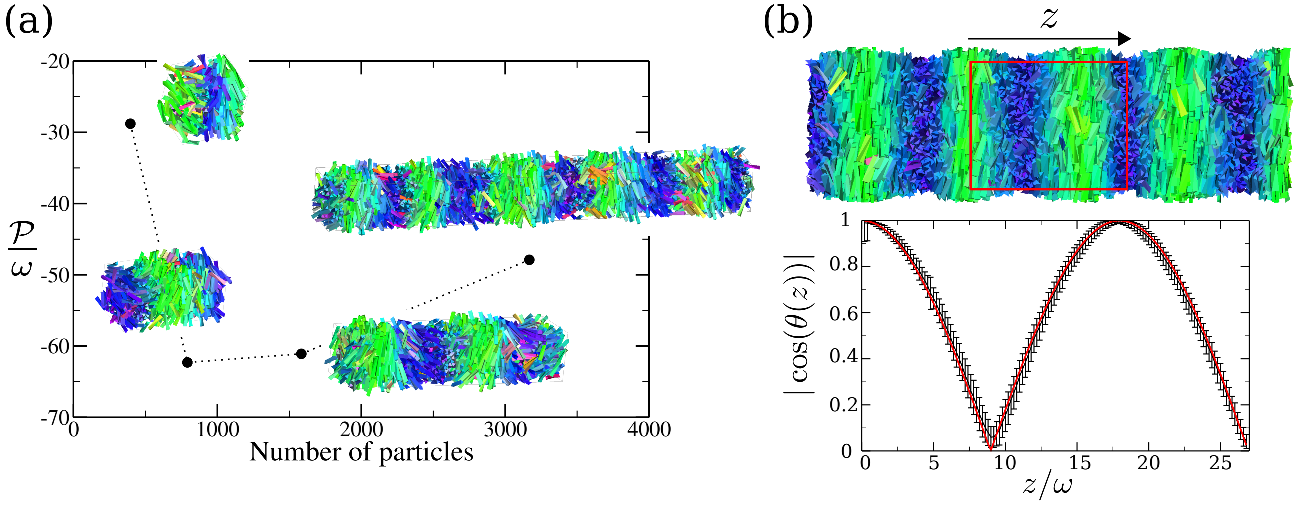

Supplementary Figure 4. **Extracting the cholesteric pitch from simulations under different boundary conditions.** (a) Cholesteric pitch $\mathcal{P}$ as a function of number of particles N as obtained from MC simulations in an NVT ensemble, but allowing the simulation box to change side dimensions while keeping the volume fixed (packing fraction $\eta \simeq 0.18$). TTP with aspect ratio $h/w = 5$, twist angle $\alpha = 0.7$, and base angle $\gamma = 1.0$. Standard periodic boundary conditions (PBC) are employed, which results in a strong dependence on the initial box size. System sizes correspond to 2,4,8 times the smallest size considered. (b) Simulations of the same TTPs, but using twisted boundary conditions. The snapshot shows the boundaries of the simulation box in red and the first periodic images. The macroscopic twist $|\cos(\theta(z))|$, with θ the twist angle along the z-direction corresponding to the chiral director. The fit used to extract the cholesteric pitch $\mathcal{P}$ is indicated with a red line (see main text for more details). Note the difference of $\pi/2$ in the θ angle at the edges of the simulation box as imposed by the twisted boundary conditions [2].

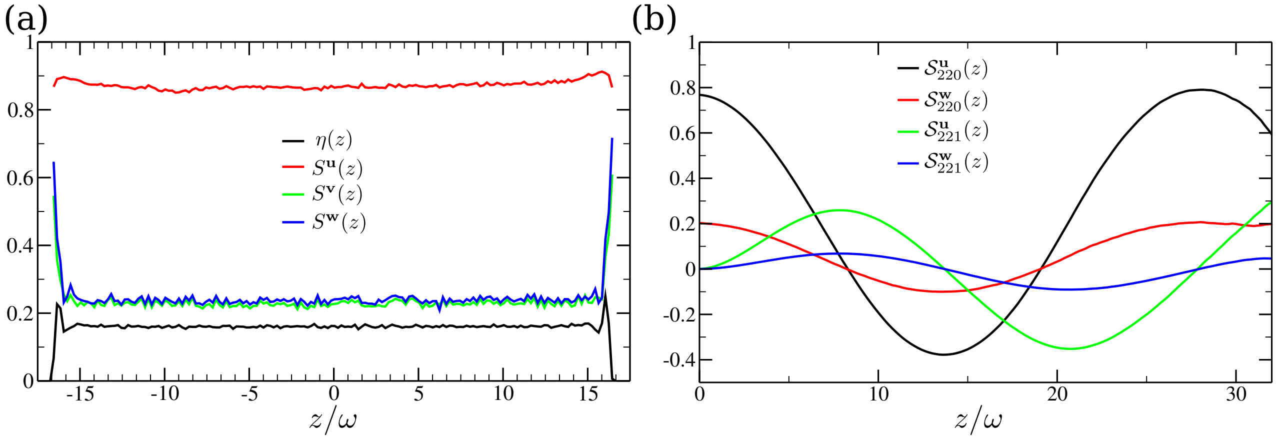

Supplementary Figure 5. **Structure of a cholesteric phase embedded between two hard walls.** (a) Packing fraction $\eta(z)$ and nematic order parameter profiles $S(z)$ for TTPs with $h/\omega = 5$, $\alpha = 0.7$ $\gamma = 0.75$ between two hard walls (situated in the z-direction) obtained by simulations in NVT ensemble ($N = 2160$, $\eta \simeq 0.16$). (b) Orientational pair-correlation functions for the same system (cfr. Supplementary Fig. 3). The helical structure is maintained but it does not need to be commensurate with the box size.

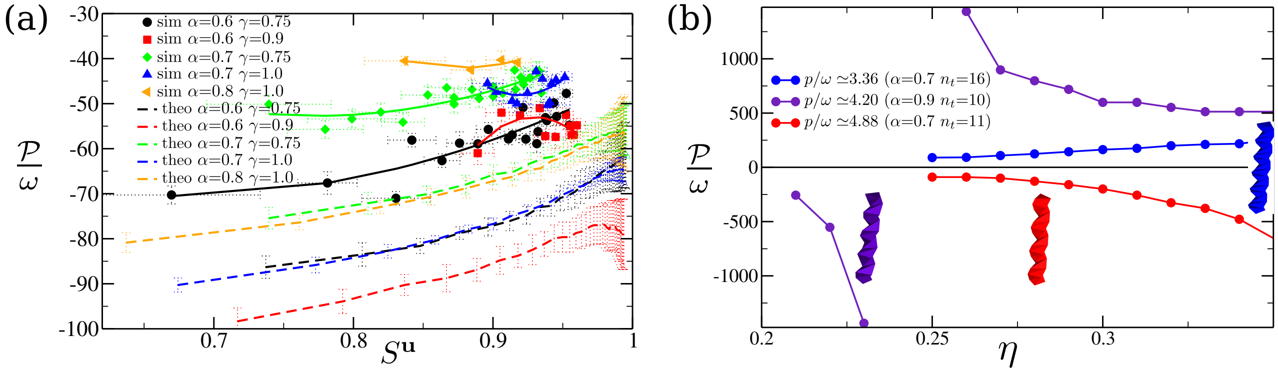

Supplementary Figure 6. **Theoretical predictions and comparison with simulations.** (a) Comparison between simulations (symbols and solid lines used as guides to the eye) and theoretical results (dashed lines) for the cholesteric pitch $\mathcal{P}$ as a function of the nematic order parameter $S^{\mathbf{u}}$ associated to the particle long axis for the same particle models considered in Fig. 5 of the main text. (b) Theoretical predictions for the cholesteric pitch $\mathcal{P}$ as a function of packing fraction η for TTP with multiple twists ("triple hard helices") with aspect ratio $h/\omega = 6$, and an equilateral triangular base ($\gamma = \pi/3$) and varying α and number of twists n_t such that the molecular chiral pitch $p/\omega \simeq 2h\pi/(n_t\alpha\omega)$ varies as indicated in the legend. Depending on p, the cholesteric pitch $\mathcal{P}$ can be positive (right-handed), negative (left-handed) or changing sign upon varying the packing fraction η (sense inversion), analogous to previous findings for hard helices [3, 4].

Supplementary References

[1] R. Memmer, Determination of equilibrium pitch of cholesteric phases by isobaric-isothermal Monte Carlo simulation. J. Chem. Phys. **114**, 8210 (2001).
[2] M. P. Allen and A. J. Masters, Computer simulation of a twisted nematic liquid crystal. Mol. Phys. **79**, 277-289 (1993).
[3] S. Belli, S. Dussi, M. Dijkstra, and R. van Roij, Density functional theory for chiral nematic liquid crystals. Phys. Rev. E **60**, 020503(R) (2014).
[4] S. Dussi, S. Belli, R. van Roij, and M. Dijkstra, Cholesterics of colloidal helices: predicting the macroscopic pitch from the particle shape and thermodynamic state. J. Chem. Phys. **142**, 074905 (2015).
